# Heart Failure and Hospital Utilization Trajectories Before and After Hip Fracture Surgery

**DOI:** 10.1093/geroni/igab046.1986

**Published:** 2021-12-17

**Authors:** Sijia Wei, Wei Pan, Chiyoung Lee, Hideyo Tsumura, Tingzhong (Michelle) Xue, Eleanor McConnell

**Affiliations:** 1 Duke University School of Nursing, Apex, North Carolina, United States; 2 Duke University, Duke University School of Nursing, North Carolina, United States; 3 University of Washington, Bothell, Bothell, Washington, United States; 4 Duke University School of Nursing, Durham, North Carolina, United States

## Abstract

Long-term hospital utilization trajectories in the context of surgery are understudied. Heart Failure (HF) is associated with an increased risk for rehospitalization after hip fracture surgery. This study aimed to examine whether older adults (>= 65 years old) have distinct patterns of long-term hospital utilization trajectories and whether HF influences these trajectories before and after hip fracture surgery. An initial cohort of 1,172 older adults hospitalized for hip fracture surgery between October 2015 and December 2018 was extracted from electronic health records. To adjust selection bias in baseline characteristics, we used propensity score 1:1 ratio matching to identify a final cohort of older adults with (n = 288) and without (n = 288) HF. Monthly frequencies of emergency department (ED) and inpatient encounters 1-year before and after the hip fracture surgery were used to identify distinct utilization trajectories from group-based trajectory analysis. Logistic regression models were used to compare the differences in ED and inpatient trajectories among patients with and without HF. High ED users (9.5%) had constant high ED use, and high inpatient users (20.1%) had significantly higher inpatient usage around the index hip fracture surgery hospitalization. Both low ED (90.5%) and inpatient (79.9%) users had low but slightly increased use around the index hospitalization. Compared with older adults without HF, older adults with HF were more likely to be long-term high inpatient user (OR = 1.94, 95% CI 1.25-3.01, p = 0.003), but not significantly different in long-term ED utilization (OR=1.87, 95% CI 0.97-3.59, p = 0.62).

